# Seasonal Influence on Pesticide Transfer and Bioaccumulation in Native Wetland Vegetation in an Agricultural Critical Zone

**DOI:** 10.1007/s00267-025-02135-z

**Published:** 2025-04-03

**Authors:** Betty Chaumet, David Riboul, Jean-Luc Probst, Pierre Eon, Anne Probst

**Affiliations:** 1 Centre de Recherche sur la Biodiversité et l’Environnement (CRBE), Université de Toulouse, CNRS, IRD, Toulouse INP, Université Toulouse 3 – Paul Sabatier (UT3), Toulouse, France; 2 LTSER Zone Atelier Pyrénées-Garonne, CNRS, University of Toulouse, 31326 Castanet Tolosan, France; 3 LTER Bassin versant Auradé, IR OZCAR, CNRS, University of Toulouse, 31326 Castanet Tolosan, France

**Keywords:** Phytosanitary product mitigation, BAF, Translocation, Pond plants, SBSE-TD-GC-MS/MS analysis

## Abstract

Wetlands are acknowledged for their significant role in mitigating contaminant fluxes to aquatic environments. However, the contribution of intrinsic vegetation to the efficacy of wetlands in dispersing pesticides remains a subject of debate. This study seeks to quantify: (1) the ability of native wetland plants to bioaccumulate pesticides in distinct compartments (roots, stems, leaves), and (2) the transfer of pesticides from sediments and water to plants, as well as within plants. Two field campaigns were conducted in a pond located in an agricultural area during two contrasting seasons: autumn and the subsequent summer. Six pesticides (metolachlor, boscalid, epoxiconazole, tebuconazole, aclonifen and pendimethalin) typical of arable farming practices and with different chemical properties were analysed in samples taken from five native plant species: *Salix alba L., Carex pendula Huds*, *Mentha aquatica L*., *Typha latifolia L*. and *Juncus inflexus L*. A new method was developed to analyse pesticides by using thermo desorption GC-MS which allowed a sensitive quantification in all plant compartments. Pesticides were found in all the plants, but *Salix alba* and *Carex pendula* proved to be the most effective accumulators of pesticides compared to other species, and showed perennial accumulation over time. The most hydrophobic molecules were mainly found in leaves, partly due to translocation. The impact of flood events, which introduced a significant amount of pesticides from the upper drainage catchment into the pond between the two sampling campaigns, was evident in terms of storage by plants. This study highlights the importance of revegetating ponds with native species as part of a wetlands remediation plan.

## Highlights

- The most efficient pesticide storage was for *Salix alba* and C*arex pendula.*
- Pesticide uptake depends on hydrophobicity; high log K_OW_ enhances root uptake, but carbonate-rich contexts may alter this trend.
- Hydrophobic molecules were mainly accumulated in the leaves.
- Flood inputs from the upstream catchment influence bioaccumulation.
- Native plants play a role in mitigating pesticides in wetlands.

## Introduction

In agricultural catchments, wetlands, such as ponds, are commonly utilized for water supplies and recreational activities. Numerous studies have emphasized their importance for biodiversity (Knight et al. 2003) and their role in regulating the influx of dissolved contaminants and suspended solids (Braskerud and Haarstad 2003; Kasak et al. 2018; Zheng et al. 2018). However, wetlands in agricultural areas face pressure from various contaminant inputs, including metals (Wu and Probst 2021), pesticides (Chaumet et al. 2021) and nitrogen in fertilizers (Wu et al. 2021). This pressure is particularly associated with flood events, which carry water and materials from soil leaching and runoff from the upper drainage catchment (Lazartigues et al. 2012; Schulz 2004).

Wetlands comprise several compartments, such as the water column, sediments, vegetation, and biota. Contaminants undergo different processes inherent to their properties within these complex and dynamic environments (Bahi et al. 2023; Rose et al. 2008), leading to a decrease in contaminant concentrations at the outlet (Imfeld et al. 2021). An example is the storage process of contaminants in vegetation through transfer and bioaccumulation (Fantke et al. 2016). Metal accumulation in vegetation is well-documented (Klink 2017; Ladislas et al. 2013, 2012; Pavlović et al. 2005), including the transfer mechanisms among different plant compartments (Probst et al. 2009).

Recent technical advances in the quantification of pesticides, mainly for health purposes (pesticide residues in food), have allowed a deeper understanding of the transfer mechanisms of these molecules. As a result, studies on the bioaccumulation of pesticides in plants and their potential contribution to the remediation process have emerged (Dhir et al. 2009; Eevers et al. 2017; Keerthanan et al. 2021). Despite these advances, few studies have addressed how wetland plants respond to pesticide contamination over time (Stehle et al. 2011). A recent study conducted by Liu et al. (2019) reviewed the mechanisms of phytoremediation for organophosphorus pesticides. They highlighted that removal efficiencies were species and compound-dependent.

Most research has focused on phytoremediation using plants with a high contaminant holding capacity (Campos et al. 2008; Paz-Alberto and Sigua 2013; US EPA 1999), overlooking native species in hazardous risk areas such as agricultural catchments (Tarla et al. 2020). Investigating the storage/bioaccumulation capacity of pesticides in each plant compartment, along with transfer factors between compartments, can enhance our understanding of plant mechanisms. This approach may identify the most efficient plants for long-term pesticide dissipation, offering a new perspective on agricultural land management to limit water pathway diffusion. Recent studies have focused on modelling the potential bioaccumulation of pesticides by plants, which represents a significant advance for large-scale land management (Li 2022; Li and Ai 2023). However, these studies have not yet included wetlands, which are complex and diverse environments that remain to be studied at this level.

As part of the ANR PESTIPOND Project (https://pestipond.cnrs.fr/), we investigated the behaviour of a pond in an agricultural (Ponnou-Delaffon et al. (2020a); Probst et al. 2024a, 2024b)context with respect to pesticides. The remediation capacity of the pond was demonstrated by studying the sediment compartment (Chaumet et al. 2021) and its ability to store pesticides depending on their hydrophobicity and the grain size distribution. In addition, an input-output mass balance over one year showed that removal rates varied depending on the molecule and the water phase (dissolved or particulate, Chaumet et al. 2022). Pesticide monitoring over one year showed the major role of flood events on pesticide inputs, which can lead to significant peaks of bioaccumulation during and after this period when the suspended particles settle.

While sediment plays a key role in pesticide retention and potential reloading, other wetland components also contribute to pesticide dissipation through a variety of processes including microbial degradation (Kang et al. 2023), photodegradation (Stangroom et al. 2000), and plant uptake (Maillard and Imfeld 2014). The rapid growth of vegetation at the pond inlet has added complexity to the ecosystem and pesticide dynamics, influencing both direct accumulation in plant tissues and indirect effects such as rhizosphere-mediated microbial degradation and sediment stabilization. As wetlands function as integrated systems where biotic and abiotic processes interact, it is essential to assess the combined roles of these compartments to develop a comprehensive understanding of pesticide mitigation and to provide practical scenarios for decision-makers in agricultural land management.

This study focuses on the development and application of a method to assess the efficiency of native wetland plants in accumulating a broad spectrum of pesticide molecules, taking into account their role within the broader wetland system. By understanding how different plant species interact with pesticides of various chemical properties, the aim is to assess the role of vegetation in pesticide dissipation and identify potential improvements to enhance mitigation processes. While wetlands function as integrated systems where vegetation, sediment, and hydrological processes collectively influence pesticide fate, understanding the specific role of plants provides critical insights into their potential to enhance mitigation strategies. The research will provide insights into how native vegetation in agricultural wetlands can be effectively used for pesticide removal, taking into account the different characteristics of the pollutants and of the hydrological and seasonal environmental conditions in the upper catchment.

Vegetation was collected during two contrasting seasons, including a period of pesticide application, in order to assess how the vegetation integrates the peaks of contamination loaded by water flow. First, pesticide concentrations and stocks were assessed in each plant compartment (roots, stems, and leaves) and in the sediment/rhizosphere to understand translocation mechanisms and bioaccumulation processes. In the second step, the variability of these processes was determined based on plant species, molecule properties, and season. To achieve these objectives, we developed a new method based on the stir bar sorptive extraction (SBSE) technique for extracting pesticides from this complex matrix. This method ensures their quantification by thermal desorption coupled with gas chromatography-triple quadrupole mass spectrometry, a highly sensitive method capable of detecting very low doses.

## Material and Methods

### Study Site

**Fig. 1:**
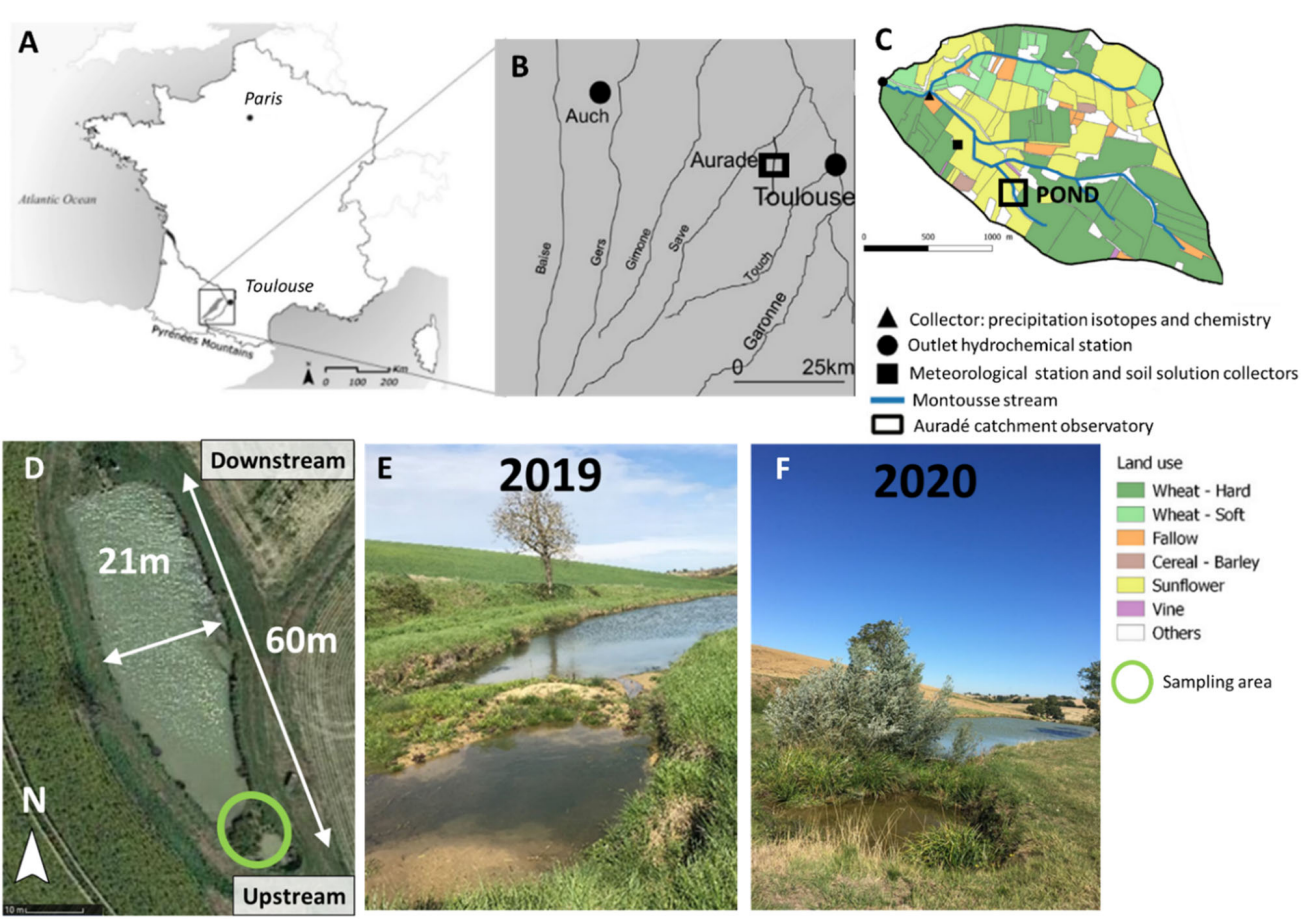
Vegetation site at the scale of **A** the country, **B** the Garonne region, **C** the Auradé catchment (adapted From Ponnou-Delaffon et al. (2020a)) and **D** Bassioué pond (from Google Maps). Pictures of the sampling site in **E** 2019 and **F** 2020. Credit photos@ A. Probst and V. Payré-Suc, respectively

The Bassioué pond is located upstream of the Montoussé catchment (328 ha) at Auradé (1°04’38.1” E, 43°33’04.7” N), in the Save basin (Fig. 1A, B) (South-West France, Gers department). The Montoussé catchment is a long-term critical zone observatory with a hydrochemical survey that has been running for 40 years (Fig. 1C; (Ponnou-Delaffon et al. 2020a; Probst et al. 2024a, 2024b).For many years, several studies have focused on assessing the impact of extensive traditional agricultural activities on water quality (Ferrant et al. 2011; Perrin et al. 2008; Ponnou-Delaffon et al. 2020b; Wu and Probst 2021) in this critical zone, which is characterized by a topography typical of the area (high slopes up to 18%) with silty-clayey soils (Gandois et al. 2011a) developed on a carbonate-rich and an impermeable molassic substratum (Probst et al. 2024c). Depending on the crop, pesticides, mainly herbicides and fungicides, are traditionally applied in this area in winter (November to March) and spring (April-May) (Taghavi et al., 2010; Chaumet et al. 2022), according to the technical information provided annually by the local farmer’s association. The steep slopes of the catchment and the occurrence of severe storm events during these periods lead to intense flash floods, which increase erosion (Ponnou-Delaffon et al. 2020b) and consequently, pose a significant risk of metals and pesticide transfer to watercourses (Roussiez et al. 2013; Macary et al. 2013).

The upstream sub-catchment (28 ha) of the Bassioué pond is well representative of the agricultural landscape of the Auradé catchment. The Bassioué pond is surrounded by cultivated zones (about 90%) with a 3-year rotation of wheat, sunflower and rapeseed. It was created in 1970 and is 60 m long and between 12 to 21 m wide. The pond was dredged in 2015, but due to its location in a highly erosive area, the sediment layer was about 2 m in 2020 and is constantly changing. The water column height is only about 50 cm, but it varies with the seasons (about 30–40 cm in summer and 50–60 cm in winter; Chaumet et al. 2022).

During the period 2015–2018, at the entrance of the pond, a sediment ridge formed as a result of sediment transport by successive significant flood events (5 m long, 7 m large Fig. 1D), particularly obvious in 2018. This sediment ridge has led to the separation of a small sub-pond in the upper part from the main pond. After 2019 (Fig. 1D), native vegetation grew very rapidly on this ridge, and in particular *Salix alba L*. (white willow), *Carex pendula Huds* (sedge), *Mentha aquatica L*. (aquatic mint), *Typha latifolia L*. (typha) and *Juncus inflexus L*. (rush) (Fig. 1E, F). Sediment texture on the ridge was predominantly coarse particles (about 65% sand and gravel), while sediment in the sub and main pond was 89 ± 2% fine particles (clay and silts). Because sediment is a major component of the pond, its role in pesticide dissipation and storage was investigated first (Chaumet et al. 2021).

For this study, we selected six pesticides with a gradient of hydrophobicity (log K_OW_): metolachlor (2.9), boscalid (3), epoxiconazole (3.3), tebuconazole (3.7), aclonifen (4.7) and pendimethalin (5.2). They were chosen because (i) four of them—metolachlor, epoxiconazole, boscalid, and tebuconazole— have been used for many years and are still used in the Auradé catchment; glyphosate was excluded due to its rapid degradation into AMPA, requiring a separate study, and (ii) aclonifen and pendimethalin, although no longer used in the catchment since 2016, persist in soils and can still be detected in sediments (Chaumet et al. 2021; 2022). The accumulation of pesticides in plants is largely influenced by their chemical properties, particularly their hydrophobicity. Pesticides with high hydrophobicity tend to accumulate more in plant tissues, especially in roots and lipid-rich areas, due to their low solubility in water (Sterling 1994). However, bioaccumulation is also influenced by other physicochemical properties, such as sorption potential (K_OC_) and persistence (half-life). Pesticides with high K_OC_ values, such as boscalid, aclonifen, and pendimethalin, bind strongly to organic matter in soils and sediments, which may limit their direct bioavailability but also create a long-term source of contamination through sediment resuspension or root uptake. In contrast, more mobile compounds such as metolachlor, with a lower K_OC_, are more readily available in the dissolved phase and may be more directly taken up by plants. Persistence also plays a role, as pesticides with longer half-lives, such as boscalid and pendimethalin, are less susceptible to degradation and may accumulate over time, whereas more rapidly degraded compounds may have lower overall availability for uptake.The carbonate-rich soils and thus sediments derived from them, can lead to alkaline conditions, and greater adsorption of pesticides into sediments, limiting their biovailability.

### Sampling Protocol and Sample Pre-treatment

Plants growing on the sediment ridge (10% of the area of the pond) were collected twice, on November 18^th^ 2019 and July 3^rd^ 2020 (Supplementary Section 1, Supplementary Fig. 1). These two sampling periods represent two key seasons for many plant species in the area (a dormant period and a growing season, respectively) and the main pesticide spreading period in the catchment (typicallin April-May), which allows to quantify the impact of pesticide peak inputs. In autumn 2019, the five main species collected were: *Salix alba L*. (white willow), *Carex pendula Huds* (sedge), *Mentha aquatica L*. (aquatic mint), *Typha latifolia L*. (typha) and *Juncus inflexus L*. (rush). In summer 2020, only the first three species were sampled because the two others were no longer present.

For each native plant species, two individuals were randomly selected from the ridge, together with their rhizospheric soil, to provide a representative sample of the site. These samples were pooled to improve spatial representation and to minimise variability between individual plants. Although we did not include multiple replicates for each species due to the high number of samples required given the limited number of plants in the area, and the significant analytical costs associated with the study, the approach still allows for a comprehensive assessment of the role of species in pesticide dissipation. Pooling the samples allowed us to focus on species-level differences rather than on individual variability, which is often less relevant for understanding general pesticide dynamics in ecological contexts. This strategy balances scientific rigour with practical constraints, ensuring that the data remain meaningful while maintaining the feasibility of the study design.

Once in the laboratory, each plant compartment (rhizosphere soil, roots, stem and leaves) was separated using a ceramic knife. *Carex pendula*, *Typha latifolia* and *Juncus inflexus* were considered as two-compartment plants as they do not have stems. The rhizospheric soil was collected by firstly removing all the soil around the roots, and secondly by gently rubbing the roots with a brush to collect only the soil near the roots. The rhizosphere soil was sieved at 63 µm to collect only the silt and clay fraction for pesticide quantification, as this is the fraction that is most abondant in the pond and has the highest sorption surface capacity (Probst et al. 1999). It is therefore more likely to influence the behaviour of pesticides in the rhizosphere (Spark and Swift 2002). Next, the roots were rinsed with demineralized water, to remove any remaining soil, as well as stems and leaves. All compartments were frozen and then freeze-dried to obtain the dry mass. Finally, each plant compartment was ground using a metal ball mill.

### Pesticide Quantification using GC-MS/MS Analysis

Pesticides were quantified by thermal desorption coupled with gas chromatography-triple quadrupole mass spectrometry (TD-GC-MS/MS (QqQ)) using the principle of internal standards. For rhizospheric soil samples, pesticides were quantified as described for soils and sediments by Chaumet et al. (2021). Briefly, the samples were dried at room temperature, crushed and sieved through a mesh to collect only the finest fraction (<63 µm). A mass of 0.5 g was placed in a 15 mL glass tube with 10 mL of methanol (purity: 99.8% for residue analysis, ACROS organics) and 100 μL of internal standard at a concentration of 80 μg.L^−1^. After extraction by sonication (vortex for 5 min - sonication for 30 min – vortex for 5 min), the samples were centrifuged for 25 min at 6000 RPM at 4 °C. Two mL of the supernatant were collected and added into a 20-mL amber glass bottle prefilled with 17 mL ultrapure water and a Stir Bar Sorptive Extraction (SBSE) bar. The pesticides were then extracted from methanol on the stir bar for three hours at 1000 RPM, before being quantified by TD-GC-MS/MS (QqQ). For the different plant organ samples, a method had to be developed to adapt the pesticide extraction protocol to the plant matrix (to avoid the matrix effect due to sugars and chlorophyll) and to our analytical detection techniques. Details of the method development are provided in the supplementary material (Supplementary Section 2).

A 1 g mass of plant polder was placed in a 10 mL glass bottle, filled with 10 mL of methanol, vortexed for 5 min at 1200 RPM, ultrasonicated for 30 min and vortexed again for 5 min at 1200 RPM. The samples were decanted for 10 min, then 2.5 mL of supernatant was collected and placed in a 15 mL Falcon® tube.

From this stage, PSA (Primary Secondary Amine) and graphitized carbon black (Thermo Scientific) were used for a d-SPE purification to remove as much residual sugar and chlorophyll as possible, respectively. Twenty-five mg of PSA was added to the samples, and they were vortexed for 5 min at 1200 RPM. Next, 50 mg of graphene was added, the tubes were vortexed again for 5 min at 1200 RPM, and then centrifuged for 25 min at 4 °C at 600 RPM. The sample’s supernatant was collected (2 mL) and placed in an amber bottle with 17 mL of MilliQ water, and with 100 µL of an internal standard stock solution (metolachlor D6 at 80 µg.L^−1^, aclonifen D5 at 160 µg.L^−1^, pendimethalin D5 at 80 µg.L^−1^, boscalid D4 at 800 µg.L^−1^ and epoxiconazole D4 at 80 µg.L^−1^, all from Cluzeau Info Labo C.I.L.). A 10-mm long stir bar coated with a 0.5 mm film thickness layer of PDMS (purchased from Gerstel) was added to the amber bottle and stirred for three hours at 1000 RPM for pesticide extraction. Finally, the magnetic bars were collected, rinsed with ultrapure water and dried before pesticide quantification by TD-GC-MS/MS.

The stir bar containing the sorbed pesticides was placed into the thermodesorption tube which was inserted into the TD-GC-MS/MS autosampler. Thermodesorption was performed using a temperature gradient with steps of 60 °C per minute from 30 °C to 280 °C, plus a stabilization step at the maximum temperature for six minutes. The sample was transferred under a 50 ml min^−1^ helium flow and focused into the CIS 4 inlet at −10 °C. Finally, the inlet was ramped to 280 °C at 600 °C min^−1^ plus a stabilization step for four minutes to transfer the pesticides into the GC column.

The chemical separation was performed by gas chromatography (TRACE 1300 Gas Chromatograph). The sample was injected into the fused-silica capillary column (30 m × 0.25 mm i.d × 0.25 μm film thickness of 5% phenyl, 95% poly-dimethylsiloxane, Thermo Scientific TraceGOLD TG-MS). The carrier gas was helium in constant flow mode at 1.2 mL min^−1^. The column temperature was programmed from 40 °C (held for 2 min) at 30 °C min^−1^ to 150 °C, at 10 °C min^−1^ to 280 °C (held for 5 min). Pesticide detection was performed with a triple quadrupole mass spectrometer (TSQ 8000 EVO, Thermo Fischer), a Thermal Desorption Unit (TDU), a Cooled Injection System (CIS 4) and MultiPurpose Sampler (MPS) to place Twister bars into the TDU system (all GERSTEL). The detection mode used was Selected Reaction Monitoring (SRM) with two ion transitions for each pesticide and Electron Impact (EI, at 70 eV). Because deuterium-labelled internal standards were added to the samples, pesticide quantification could be performed by an internal calibration (details provided in Supplementary Section 2, Supplementary Tables 1 and 2), the calibration curve was also performed using the SBSE stir bar. The data processing was done by TraceFinder software (Thermo Scientific version 3.3).

### Data Processing

Pesticide stocks (in ng) in every compartment (i) was calculated as follows:

1 $${{Mass}}_{{pesticides}-{compartment\,i}}={\left[{pesticide}\right]}_{{compartment\,i}}* {{Mass}}_{{compartment\,i}}$$

Where ${[{pesticide}]}_{{compartment\,i}}$ is the pesticide concentration in the plant compartment i, in µg.kg^−1^ and ${{Mass}}_{{compartment\,i}}$ is in the dry biomass weight of the plant compartment i, in g.

The Bioaccumulation Factor (BAF) is unitless and was calculated as follows (Eq. (1)):

2 $${BAF}=\frac{{[{pesticides}]}_{{Plant}}}{{[{pesticides}]}_{{rhizospheric\,soil}}}$$

with the pesticide concentration (in µg.kg^−1^) in the whole plant (numerator) and the pesticide concentration in the rhizospheric soil (denominator, in µg.kg^−1^).

A BAF greater than 1 indicates a bioaccumulation of the pesticide for the whole plant.

The translocation of pesticides from one compartment to another was estimated using TF (Eq. (2)). TF was calculated as the ratio between pesticide concentrations in two plant compartments following Eq. (2).

3 $${TF}=\frac{{[{pesticides}]}_{{compartment\,in}}}{{[{pesticides}]}_{{compartment\,ext}}}$$

with the pesticide concentration in *compartment in* (the compartment where the pesticides go) and in *compartment ext* (the compartment that the pesticides come from) expressed in µg.kg^−1^. A TF greater than 1 means that the pesticides were transferred from a given storage compartment to a receiving compartment.

## Results

### Vegetation Characterization

All five plant species have a typical continental habitat (terrestrial and/or freshwater). The flowering period extends from April to September, specifically from April to May for *Salix alba*, from May to July for *Carex pendula*, from July to September for *Mentha aquatica* and from June to September for *Typha latifolia* and *Juncus inflexus*. The root system of *Salix alba* was highly developed, as this species is known for its tap roots and tracer roots, which can penetrate into the substrate (from 10 to 50 cm according to field observations) and represented 25–32% of the total plant mass (see details in Supplementary Section 1). In *Carex pendula, Mentha aquatica* and *Juncus inflexus*, the roots were quite long compared to the leaves (2/3 of the length) and represented 27, 64 and 14% of the plant mass, respectively. This is in contrast to *Typha latifolia*, which had very short roots (10–20 cm), consisting of a single thick root that represented 36% of the total plant.

For *Salix alba*, for both seasons, stems represented the largest compartment in terms of weight, about twice as much as the roots. The opposite proportion was found for *Mentha aquatica* for which the root compartment represented between half and ¾ of the weight of the whole plant (Table 1). For *Carex pendula*, *Typha latifolia* and *Juncus inflexus*, leaves were the main compartment (accounting for between 64 and 86% of the total biomass). When comparing autumn to summer, no substantial difference was observed in terms of biomass proportion between compartments for *Salix alba* and *Carex pendula*. Conversely, for *Mentha aquatica*, the root proportion decreased in favour of the stem and leaf compartments from fall to summer.

**Table 1:** Plant mass distribution in % by compartment and season based on the dry mass presented in Supplementary Table 4

		Plant compartments mass distribution (%)		
		Roots	Stems	Leaves
*Salix alba*	Autumn	25.9	60.8	13.3
	Summer	31.9	51.6	16.5
*Carex pendula*	Autumn	21.3	–	78.7
	Summer	31.9	–	68.1
*Mentha aquatica*	Autumn	75.6	14.5	9.9
	Summer	52.4	24.6	23.0
*Typha latifolia*	Autumn	35.7	–	64.3
*Juncus inflexus*	Autumn	13.9	–	86.1

### Pesticide Concentrations and Distribution within the Plant and the Rhizosphere

**Fig. 2:**
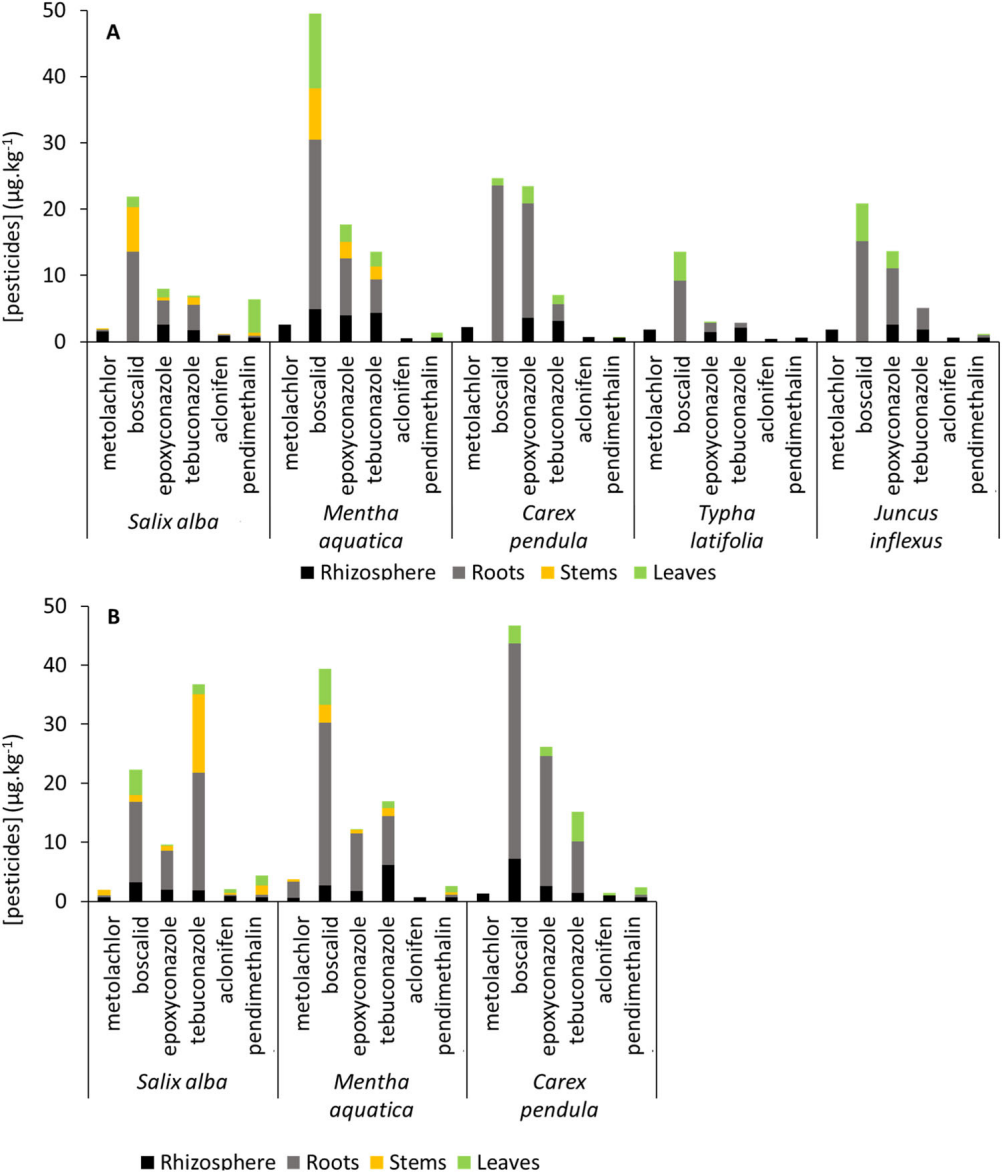
Pesticide concentrations in the rhizosphere and the different compartments of each vegetation species in µg.kg^−1^, for **A** Autumn and **B** Summer

For all plants, the highest concentrations of pesticide were found in roots, except for pendimethalin, which was more often concentrated in leaves, especially for *Salix alba* (Fig. 2). Boscalid was the most concentrated pesticide regardless of plant species and season (except *Salix alba* in summer) with concentrations ranging from 1.2 to 25.6 µg.kg^−1^ in autumn and 1.2 to 36.5 µg.kg^−1^ in summer (Fig. 2). Metolachlor and aclonifen concentrations were slightly above the detection limits in the different plant compartments, especially in autumn. Except for these two molecules, the general concentration trend in the plants was as follows: boscalid > epoxiconazole > tebuconazole > pendimethalin. For the three species common to both sampling campaigns, pesticide concentrations in plants were slightly higher in summer than in autumn.

All pesticides were detected in the rhizosphere of all plants, except boscalid in autumn which was detected only for *Mentha aquatica* rhizosphere (4.9 µg.kg^−1^). The highest concentrations were detected in autumn for boscalid and tebuconazole in the rhizosphere of *Carex pendula* and *Mentha aquatica*, respectively. During the same periods, the same pesticides were quantified in the sediments of the Bassioué pond and the concentrations ranged from 0.6 to 28.1 µg.kg^−1^ (Chaumet et al. 2021), which is in agreement with the concentrations observed in the plants, as they mainly accumulated from the sediments. In general, the pesticide concentrations measured in vegetation in this study were rather low compared to those that *Typha Latifolia* is capable of bioaccumulating (from a few µg.kg^−1^ to several hundred µg.kg^−1^ in microcosms, Pérez et al. 2022).

### Stock of Pesticides and their Distribution

**Fig. 3:**
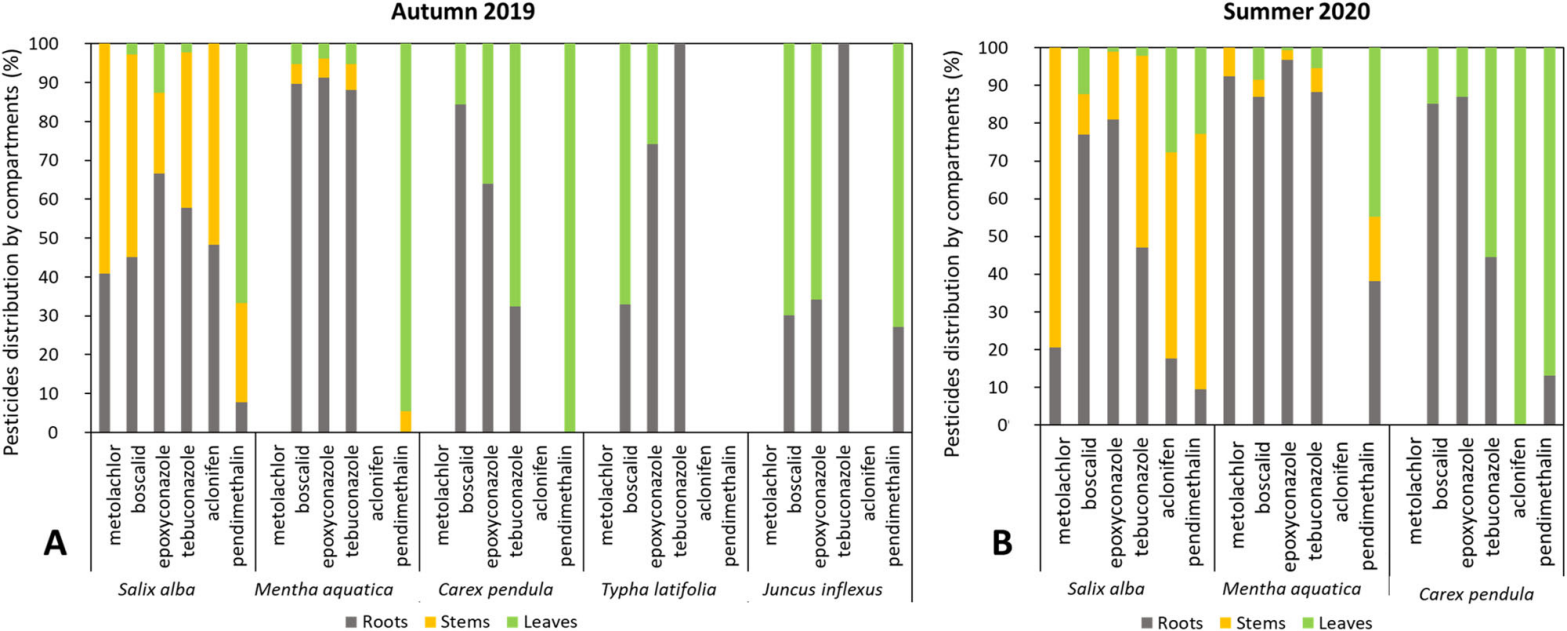
Relative distribution of pesticide stocks in roots, stems and leaves of the plant species: **A** Autumn 2019 and **B** Summer 2020. Metolachlor and aclonifen are not always presented due to negligible concentrations

The distribution of pesticide stocks in the plant compartments depended on the molecules and the plant species considered (see the relative distribution in Fig. 3; and absolute data are presented in the Supplementary Section 3, in Supplementary Table 5). From autumn to summer, tebuconazole stocksexpressed in absolute mass increased in *Salix alba* and *Carex pendula* by 3.5 and 4 times respectively, while boscalid increased threefold only in *Carex pendula* (Supplementary Section 3, in Supplementary Table 5). As a whole, the distribution of root > stem > leaves was consistent with Hosseini Alhashemi et al. (2011). Regardless of the season, pesticide stocks were most often higher in roots and stems, except for pendimethalin, which was preferentially accumulated in leaves (66% in autumn for *Salix alba*). In *Carex pendula*, an increasing proportion of pesticide storage was observed in leaves from boscalid to pendimethalin, for both conditions. In *Mentha aquatica*, roots were the highest storage compartment for all the molecules, except for pendimethalin, which was completely stored in leaves in autumn, whereas in summer it was more evently distributed between roots (38%), stems (17%) and leaves (45%). For *Typha latifolia* and *Juncus inflexus*, tebuconazole was totally stored in roots (Fig. 3).

The respective relative distribution of pesticides stocks in the different compartments of *Carex pendula* and *Mentha aquatica* (except for pendimethalin) was similar in autumn and summer, in contrast to *Salix alba* where an increase was observed in stems towards roots and leaves (except boscalid and epoxyconazole; Fig. 3). In autumn, more than 90% of all the pesticides were in leaves (the rest in stems), while in summer this proportion decreased to 44% in favour of roots.

### Bioaccumulation Factor (BAF) and Transfer Factor (TF)

*Typha latifolia* and *Juncus inflexus* did not accumulate any pesticides, except for epoxiconazole in the latter (BAF = 1.4). In summer, BAF > 1 were numerous (three to four of the six target molecules) and higher than in autumn when only *Mentha aquatica* had high BAFs for a set of molecules. Although BAFs do not reach the highest values, epoxiconazole is the most frequently bioaccumulated pesticide, followed by boscalid, which has the highest BAFs with tebuconazole (6.2 and 7.6 for *Salix alba* and *Mentha aquatica* in summer, respectively, Table 2).

**Table 2:** BAF (Bioaccumulation factor) of pesticides for the whole plant calculated for both seasons and each plant species

	Autumn					Summer		
	*Salix* *alba*	*Mentha* *aquatica*	*Carex* *pendula*	*Typha* *latifolia*	*Juncus* *inflexus*	*Salix* *alba*	*Mentha* *aquatica*	*Carex* *pendula*
Metolachlor	0.1	0.0	0.0	0.0	0.0	0.9	**2.6**	0.0
Boscalid	0.0	**4.4**	0.0	0.0	0.0	**1.8**	**6.2**	**1.9**
Epoxiconazole	0.5	**1.8**	**1.6**	0.5	**1.4**	**1.3**	**3.0**	**3.1**
Tebuconazole	**1.0**	**1.0**	0.5	0.1	0.3	**7.6**	0.8	**4.3**
Aclonifen	0.1	0.0	0.0	0.0	0.0	0.4	0.0	0.3
Pendimethalin	**1.7**	0.1	0.2	0.0	0.4	**1.8**	0.8	**1.4**

BAF higher than or equal to 1 are in bold

**Fig. 4:**
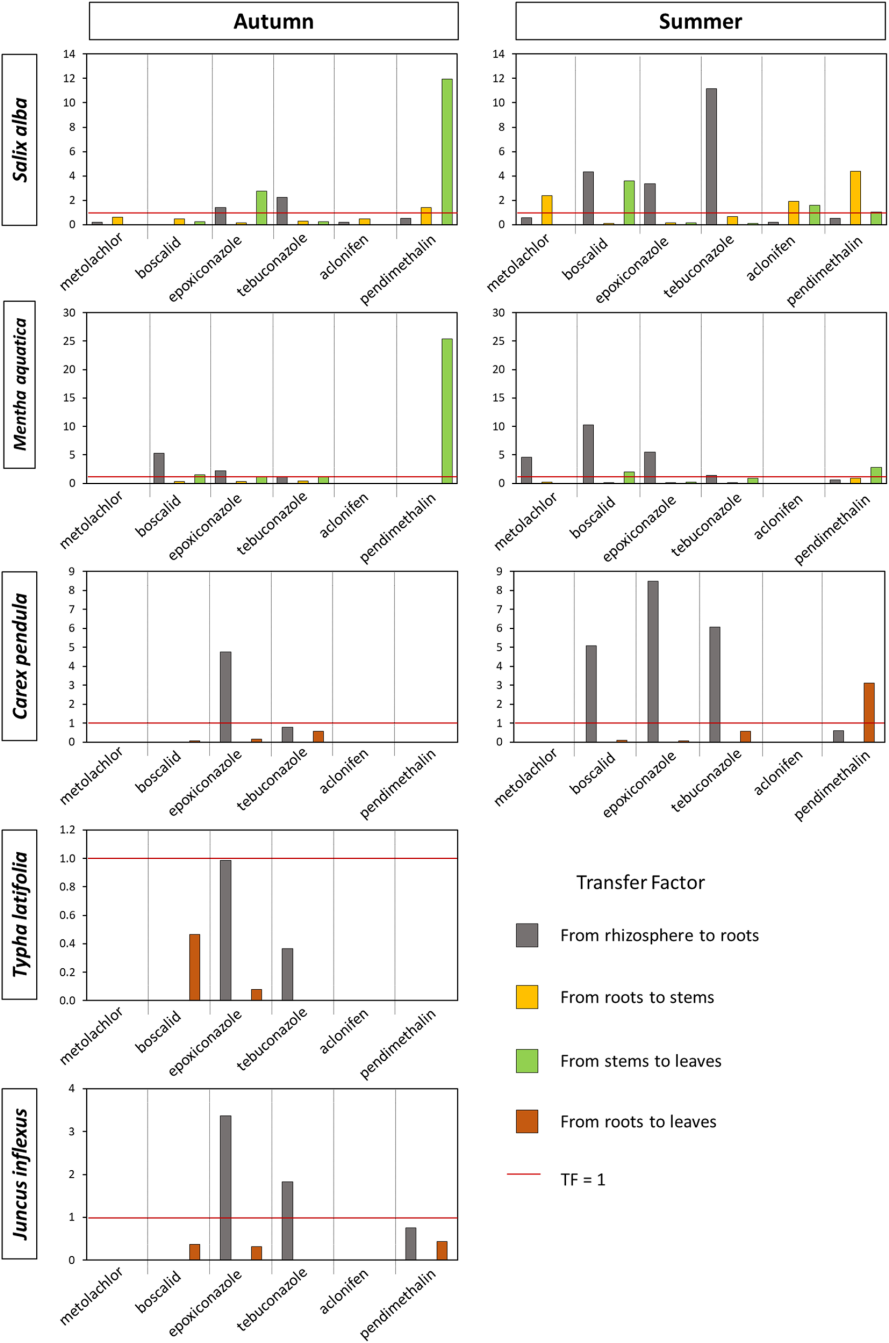
Pesticide translocation (TF) for each plant and season

Overall, the most obvious pesticide transfer (TF ≥ 1) was from the rhizosphere to the roots (Fig. 4), which is particularly evident for epoxiconazole (except in *Typha Latifolia*). Metolachlor transfer was not -or almost not- quantifiable in *Carex pendula*, *Typha latifolia*, *Juncus inflexus*, *Mentha aquatica* and *Salix alba* (except for a limited transfer to roots and stems in summer for the latter).

*Salix alba* had a TF that was most often greater than 1 for most compartments and for both seasons. More specifically, the highest TFs were from stem-to-leaf in autumn for epoxiconazole (2.8) and pendimethalin (11.9). In summer, the TF from rhizosphere to roots exceeded 1 for boscalid, epoxiconazole and tebuconazole, while a preferential translocation pathway was observed from stem to leaf for aclonifen and pendimethalin. Very similar patterns were observed for *Mentha aquatica* in summer and TFs were more often equal to or greater than one in autumn, with in particular the highest TF for stem to leaf translocation for pendimethalin (25.4, Fig. 4).

For *Carex pendula*, only the bioaccumulation from rhizosphere to root was noticeable (TF > 1), especially in summer for boscalid, epoxiconazole and tebuconazole. For *Typha latifolia and Juncus inflexus*, the TF patterns were similar (TFs < 1), except from rhizosphere to root for epoxiconazole and tebuconazole for *Juncus inflexus*. These data are within the range of data from Haller et al. (2018) who reported TFs = 0.1–10.8 from roots to stems and TFs = 0.1–25.7 from roots to leaves.

## Discussion

### Role of Season and Hydrological Conditions on Bioaccumulation in the Whole Plant

The bioaccumulation of pesticides has been established with precision thanks to the method developed for these samples involving thermo-desorption (see details in SI). This new method provides a more sensitive analysis by eliminating the matrix effect, enabling the identification of substances that might otherwise have gone undetected.

Bioaccumulation is a reversible storage process in plants, subject to factors such as plant death and leaf fall. When plants senesce, predominantly in autumn, stored contaminants may be released into the environment. Over time, the most resilient plants in the habitat may replace more sensitive species. This likely occurred between autumn 2019 and summer 2020 for *Typha latifolia* and *Juncus inflexus*, which disappeared from the sediment ridge. Indeed, flood events between the two sampling periods altered the shape of the sediment ridge, pushing *Typha latifolia* further away. Conditions became less favourable for these species (see more details on the impact of the habitat on the vegetation distribution in Supplementary Section 1), despite their detoxification abilities (Dhir et al. 2009).

Between the two field campaigns in autumn 2019 and summer 2020, another parallel study was carried out on this wetland to estimate pesticide inputs and outputs (Chaumet et al. 2022). During this period, a major flood event occurred in May 2020, contributing 493 mg of tebuconazole to the pond. The input of the other molecules was not substancial. From autumn to summer, there was a noticeable increase in the stock tebuconazole in *Salix alba* and *Carex pendula* which was certainly linked to large inputs of pesticides. It is possible that the high pesticide input during this flooding period induced a toxic effect in *Typha latifolia* and *Juncus inflexus*, which could also have contributed to their disappearance from one season to the next. The slight difference in tebuconazole concentration observed between seasons could be attributed to plant growth during the spring and summer of 2020, increasing storage capacity. For example, the growth of *Salix alba* led to almost constant pesticide concentrations, indicating that there was no dilution effect but a rather constant accumulation *via* translocation mechanisms between compartments. The impact of pesticide inputs during flood events is evident in the increase in the bioaccumulation factor (BAF) of boscalid in plants between autumn and the following summer, despite the increase in stocks for *Carex pendula*.

### Role of Molecule Characteristics on Bioaccumulation and Transfer of Pesticides

Roots are a preferential route for contaminants to reach the upper compartments (Kaur et al. 2024). The pesticides targeted in this study were more likely to have been absorbed passively because they are both neutral and lipophilic, and are therefore easily transported to lipidic membranes (Sterling 1994). Pesticide uptake depends, among other factors, on the hydrophobicity of the substance (Zhang et al. 2017): a high log K_OW_ increases uptake by the roots. Except for metolachlor, which is rarely detected in the water throughout the year, the decrease in concentration observed in the roots with an increase in the log K_OW_ of the molecules contradicts this principle as well as the observations of Wang and Kelly (2017) and Logan and Birkett (2007). This discrepancy may be due to the carbonate context of the Bassioué pond (Gandois et al. 2011b; Perrin et al. 2008). These findings suggest that pesticides such as boscalid are effectively sequestered in the root compartment.

The smectite-type clay sediments of the pond, which are known to retain contaminants better in carbonate contexts (Wu et al. 2021), may explain why most hydrophobic compounds do not have high concentrations in the roots. Hydrophilic pesticides, such as metolachlor, remain predominantly in the dissolved phase in water, making them more bioavailable for root uptake via the symplastic pathway, where they move through cells and plasmodesmata (Zhang et al. 2017). These compounds are more likely to be translocated to aerial plant tissues via xylem transport due to their higher solubility in sap (Li 2022). In contrast, hydrophobic pesticides, such as pendimethalin and aclonifen, tend to adsorb to organic matter and sediment particles,- especially in a carbonate context-, limiting their mobility in water. Their uptake is mainly by the apoplastic pathway, where they passively diffuse across lipid-rich membranes and accumulate in root tissues (Sterling 1994).

Hydrophilic molecules, mainly in the dissolved phase, are available for bioaccumulation in vegetation, and studies have shown their higher sorption rates in plants than in sediments (Moore et al. 2013; Rogers and Stringfellow 2009; Tang et al. 2017). Hydrophobic molecules, mainly adsorbed onto particles, may face competition between their affinity for sediment and vegetation. However, no clear relationship between BAF or TF and the hydrophobicity of the compounds could be demonstrated. Olisah et al. (2021) came to the same conclusions about the absence of a clear relationship between bioaccumulation and hydrophobicity. They studied even more hydrophobic compounds such as PAHs and suggested that other properties such as the solubility of the compounds and the K_OC_ might be involved in the uptake process. Indeed, Li (2022) showed that it is more difficult for hydrophobic compounds to be transferred to the upper parts of the plants due to their low solubility in xylem sap. In the current study, we observed that pendimethalin, the most hydrophobic compound in the studied set, was strongly present in the leaves, which suggests according to the literature that another sorption route has been used such as direct absorption (foliar spray).

In addition, the spatial distribution of pesticides in the sediment of the Bassioué pond, as reported in a previous study Chaumet et al. 2021), further supports these observations. This study showed that pesticide concentrations were generally higher at the inlet of the pond, where partice-bound contaminants were stored in the sediment compartment due to sedimentation processes. This distribution is consistent with the properties of the 6 pesticides investigated (K_OC_ and K_OW_). Hydrophobic pesticides such as pendimethalin and aclonifen, which are highly adsorbed to sediments, were mainly retained at the inlet, reducing their bioavailability for root uptake. In contrast, more soluble compounds such as metolachlor, which have a lower affinity for sediments, remained more available in the water column and thus more accessible to vegetation. As the sampled vegetation is located at the inlet of the pond, it is likely to be exposed to higher sediment-bound pesticide loads, which may explain why some hydrophobic compounds accumulated less in plant roots than expected. These resultsreinforce the idea that both sediment dynamics and contaminant properties play a crucial role in pesticide bioavailability and uptake pathways in wetland vegetation.

### Role of Plant Species and Compartments on Bioaccumulation and Transfer of Pesticides

#### Bioaccumulation capacities

Our data underscore the significant role of the rhizosphere as a storage space for pesticides before root absorption. Concentrations in the rhizosphere were non-negligible compared to those in the entire plants, signifying its substantial role as a storage space for pesticides before root absorption.

The main accumulation of pesticides observed in the roots was to be expected, given that most pesticides are generally absorbed by the roots (pre-emergence) (Zhang et al. 2017). Pendimethalin is an exception, as it can also be easily absorbed by the leaves following foliar spraying (US EPA 1996). Pendimethalin is also highly persistent, with a half-life ranging from 3 months to 2 years, which likely explains its high concentrations in leaves compared to other compartments. Pesticide concentrations in the roots are negatively correlated with root depth. Although *Salix alba* has the longest roots, pesticide concentrations were lower than those of other plants whose roots were more concentrated at the surface. In addition, *Salix alba* has tap roots which have a smaller sorption surface. The roots of *Mentha aquatica* represent between 50 and 70% of the total mass of the plant and are widely spread horizontally on the surface. The permanent contact with fresh sediments (on the surface), which are highly loaded with contaminants, could explain the high concentrations found in this compartment.

*Mentha aquatica* displayed consistent pesticide concentrations, particularly in roots, likely influenced by continuous contact with clayey sediment known for adsorbing contaminants (Leleyter and Probst 1999; Weber et al. 2004). As it is semi-immersed, *Mentha aquatica* offered a direct contamination pathway through the entire plant. The prominence of stems and leaves over roots during the summer growing season might have contributed to higher storage for metolachlor and pendimethalin. The low bioaccumulation factor for *Typha Latifolia* could be due to its root system which is known to be large and with tough cell wall architecture limiting the translocation of substances to higher compartments (Ebrahimbabaie et al. 2023).

Variations in bioaccumulation factors between species may have limitations, as species with low accumulation rates may also have high rates of release or desorption. This may explain the low ratios between whole plant and rhizosphere concentrations (Passeport et al. 2011; Pignatello 1999; Vallée et al. 2016). These values can also be influenced by the presence of biofilms acting as a pre-barrier in the roots, stems or leaves of submerged plants.

#### Pesticide transfer in the plant

Our results highlight the time-dependent storage of pesticides in leaves, observed through the increase in translocation factors from roots to leaves, emphasizing the potential contribution of *Salix alba* to the storage and dissipation of pesticides on the time scale of the wetland.

The slight increase in the relative proportion of pesticides in leaves, along with an increase in TF from roots to leaves, may suggest time-dependent storage in leaves. Similar findings were observed by Pérez et al. (2022) for the distribution of atrazine in *Typha latifolia*, where leaves were identified as higher accumulators compared to other compartments.

*Salix alba* shed its leaves between fall 2019 and summer 2020, potentially eliminating some pesticides accumulated during the year. A high translocation factor in summer from stems to leaves indicated pesticide migration to plant tips, while autumn fluxes remained stagnant meaning that the fluxes in autumn might be frozen, or volatilized or degraded as suggested by Li (2020) and Qu et al. (2018). This trend was consistent for epoxiconazole, tebuconazole, and boscalid in *Carex pendula* and *Mentha aquatica*, with higher translocation factors in summer 2020 than in autumn 2019, notably in the transfer from the rhizosphere to the roots.

In autumn, the distribution of boscalid was roughly 50:50 between roots and stems, which implies a balance between these compartments. The following summer, this ratio shifted in favour of leaves and roots, highlighting the role of the cycle of native plants. The release of contaminated leaves into the environment resulted in significant re-absorption of rhizosphere pesticides after winter, coinciding with the growth of new leaves. This raises questions about the efficiency of plant storage over time. While a new sampling in fall 2020 could reveal a similar trend, pesticide concentrations in leaves were substantially lower than in other compartments, suggesting that *Salix alba* can store pesticides over time and contribute to their dissipation at the wetland scale.

### The Interest of Native Vegetation in Contaminated Ponds

Vegetation participates in the attenuation of pesticides in the Bassioué pond through bioaccumulation processes. The same (or even slightly lower) range of pesticide concentrations was found in the whole plants than in the pond sediment (0–30 µg.kg^−1^, for the six compounds, Chaumet et al. 2021). These values testify to a bioaccumulation process in plants, which is equivalent to storage in the sediment. However, the storage capacity of the plants is lower than that of the sediment since the sediment layer is about 1.5–2 m in the whole pond. From a practical point of view, to improve the pond efficiency, it is currently easier to manage a wetland by increasing the vegetation cover than by dredging the sediments (which are untreated waste).

Vegetated wetlands are known to be effective in pesticide mitigation (Maillard et al. 2011; Stehle et al. 2011), as plants can be effective solutions for site remediation, absorbing and transforming organic contaminants to some extent (Dhir et al. 2009; Eevers et al. 2017; Keerthanan et al. 2021; Vymazal and Březinová 2015). Plants can use different pathways to dissipate pesticides, either through rhizospheric microbial degradation or through phytotransformation (Schwitzguébel et al. 2006). Typha latifolia and Juncus inflexus were either inefficient accumulators or excellent biodegradators. As pesticide concentrations were higher in the rhizosphere than in the plant itself, one explanation could be that the pesticides did not reach the upper compartments or that the substances were biodegraded. *Typha latifolia* is particularly well-known for phytoremediation (Lv et al. 2016; Moore et al. 2017; Pérez et al. 2022), which could explain the low pesticide concentrations in the upper compartments.

For pesticide dissipation, vegetation can be considered as a barrier or filter since its roots can retain sediments and thus increase hydrological retention time (Maillard et al. 2011) giving time for pesticide bioaccumulation or avoiding resuspension (Braskerud 2001). In the Bassioué pound, vegetation has settled naturally by forming the sediment ridge which greatly enhances the dissipation of pesticides through a combination of speed reduction and accumulation in the rhizosphere and bioaccumulation (Dhir et al. 2009). For example, the establishment of vegetated islands for breaking the water flow speed seems to be a promising solution. Moreover, even some terrestrial plants seem to acclimatise perfectly to this kind of environment such as *Salix alba* also well known to be effective in phytoremediation for atrazine, metolachlor, diazinon, dimethoate, dicamba, trifloxystrobin, tebuconazole, metalaxyl, trifluralin, and nitrate (Kumar et al. 2018). In contrast, research by Warsaw et al. (2012) did not indicate any influence of *Salix alba* in the remediation of metalaxyl and trifluralin. These observations indicate that the storage and degradation of pesticides in vegetation may depend on the species considered, but also on the context of the site (for example: nursery bed *vs*. arable lands).

To date, the policy of remediating sites using the phytoremediation approach is more developed for soils but still requires further improvement and research (Fermeglia and Perišić 2023). Our study highlighted that in-situ vegetation, especially *Salix alba* and C*arex pendula*, in riparian wetlands can be a real asset in pesticide dissipation. This study could contribute to the development of a plan to clean up watercourses by using wetlands and native vegetation. Larger-scale studies would be needed to establish a solid plan that could be included in the regulations governing contaminated sites. The role that the plant compartment plays in a wetland regardless of its complexity in terms of species variety (maximum 5 major species in the case study), was demonstrated. The five plants studied were effective in storing pesticides and participating in the overall phytoremediation of the site to some extent. Even if they were not sufficiently effective to be considered as environmental purifiers as in the study of Mercado-Borrayo et al. (2015), they were effective in regulating the transfer of contaminants and the storage of pesticides.

Finally, all kinds of plants (aquatic or terrestrial) can play a non-negligible role in the retention of pesticides, especially in agricultural areas. Some improvements could be made to ensure a greater storage capacity: for example, the vegetation cover in the pond could be increased by creating vegetated islands in the water path. A combination of several species could increase the range of pesticides absorbed and stored or degraded, and would also increase the microbial diversity needed in the rhizosphere to biodegrade pesticides. For permanent dissipation, it would be preferable to promote plant species that lose only a small part of their biomass in autumn and winter. Wetlands dedicated to pesticide dissipation can be entirely landscaped with plants known to be effective in degrading pesticides while taking into account the ecology of each plant according to geography. Some examples can be found in Vymazal (2013). However, using plants for pesticide dissipation in an area with a high risk of contamination, such as an agricultural critical zone, will only reduce the toxicological risk in the watercourse, and will not eliminate it completely as the pesticide input could be too great for the plant’s storage capacity. Given that in these agricultural areas, pesticide inputs are still fairly high and continuous, other upstream solutions should be considered for long-term pesticide mitigation, including the search for pesticide substitution solutions.

## Conclusion

Our study aimed to assess the bioaccumulation and translocation of six pesticides in various vegetation compartments within a pond situated in an agricultural context during two distinct seasons. The SBSE-TD-GC-MS/MS method was successfully used to map contamination by hydrophilic and hydrophobic pesticides in plant compartments. Our findings unveiled that bioaccumulation hinged on the plant species, the specific pesticide molecule, and was notably influenced by upstream land use. Important differences in pesticide accumulation were observed between plants, and not all the plants were present in both seasons studied. A seasonal effect was observed linked to the application of pesticides upstream, but also to the occurrence of major flood events. The most efficient pesticide storage for the whole plant was found for *Salix alba* and C*arex pendula*. The roots seemed to be the primary compartment for accumulation. However, pendimethalin stood out in the leaf compartment of various species, signalling an important translocation phenomenon across the entire plant.

Furthermore, our results indicate that even a limited plant system may play a role in the dissipation of pesticides. Although pesticide concentrations found in plants were relatively low, they were of the same order of magnitude as concentrations in sediments, underscoring the significance of bioaccumulation in retaining organic contaminants. Ultimately, this study highlights the positive impact of the presence of native vegetation to mitigate pesticides in areas susceptible to considerable anthropogenic pressures. However, this should not prevent us from looking for alternatives to limit pesticide inputs to upstream land surfaces.

## Supplementary information

Supplementary information

## Data Availability

Data are provided within the manuscript or supplementary information files.
