# Supplementary material for: Seasonal Influence on Pesticide Transfer and Bioaccumulation in Native Wetland Vegetation in an Agricultural Critical Zone
Source: Environ Manage. 2025 Apr 3;75(5):1139–54. doi: 10.1007/s00267-025-02135-z (PMC12033114; doi:10.1007/s00267-025-02135-z)
Supplement: Supplementary file 1 — Supplementary information [file 267_2025_2135_MOESM1_ESM.docx]

**Supplementary information**

**Seasonal Influence on Pesticide Transfer and Bioaccumulation in Native Wetland Vegetation in an Agricultural Critical Zone**

Betty Chaumet^1,2,3^*, David Riboul^1^, Jean-Luc Probst^1,2,3^, Pierre Eon^1^, Anne Probst^1,2,3^*

^1^ Centre de Recherche sur la Biodiversité et l'Environnement (CRBE), Université de Toulouse, CNRS, IRD, Toulouse INP, Université Toulouse 3 – Paul Sabatier (UT3), Toulouse, France

^2^ LTSER Zone Atelier Pyrénées-Garonne, CNRS, University of Toulouse, 31326 Castanet Tolosan, France

^3^ LTER Bassin versant Auradé, IR OZCAR, CNRS, University of Toulouse, 31326 Castanet Tolosan, France

* Correspondence: betty.chaumet@toulouse-inp.fr (B.C.); anne.probst@toulouse-inp.fr (A.P.)

# **Habitat and vegetation distribution**


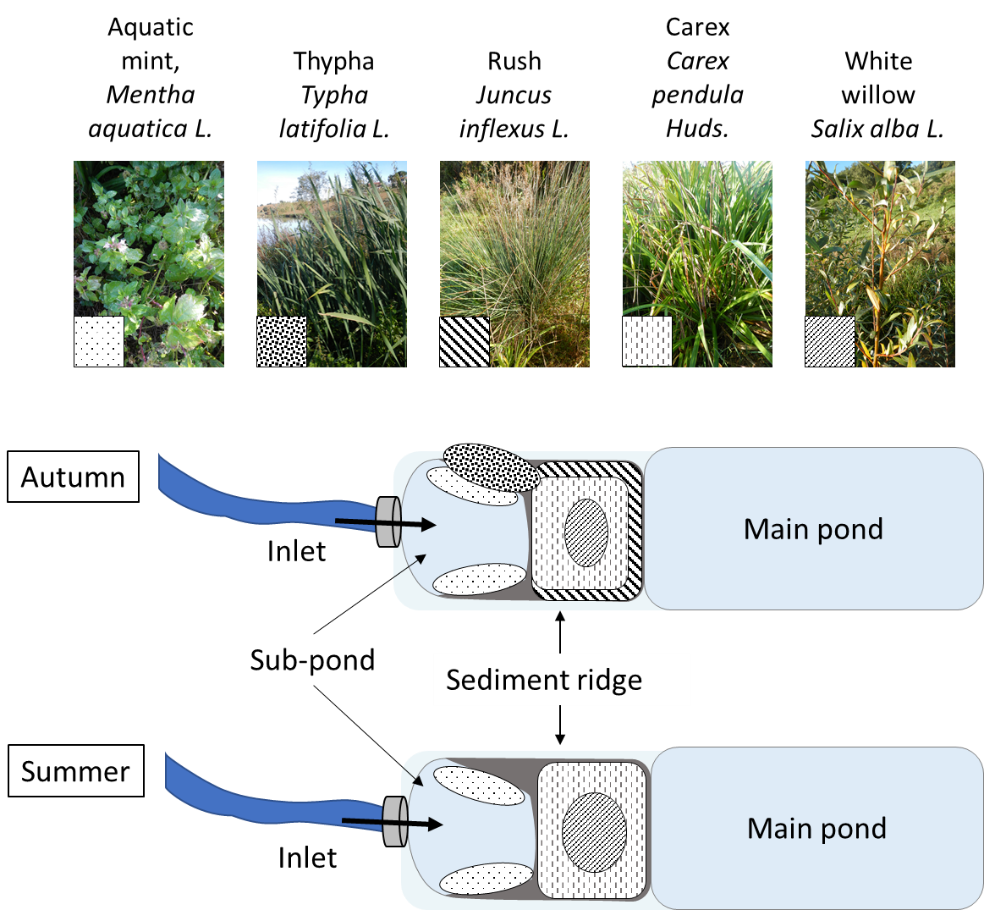


**Fig. SI 1** Schematic plant location on the sediment ridge in summer and autumn periods. Note that the scale is not respected. The vegetation on the ridge represents only 10% of the whole pond area.

In general, each plant has a specific habitat regarding water and soil characteristics. In the Bassioué pond, a gradient of sediment texture was observed, ranging from clayey sediment (in the “sub-pond” and in the main pond) to sandy soil in the middle of the sediment ridge (see Chaumet et al. 2021). *Salix alba* was found in the middle of the sediment ridge, *Carex pendula* and *Juncus inflexus* were on the ridge but on the border, and *Mentha aquatica* and *Typha latifolia were* on the side of the sub-pound (Fig. SI 1). Their location is explained by their difference in affinity with clay and their water needs. For example, *Salix alba,* as a fully terrestrial plant, can capture water down to the water table through its root system (Landgraf et al. 2021). *Carex pendula* and *Juncus inflexus* grow in wet areas but never completely submerged (Brändel and Schütz 2005; Norman 2012) in contrast to *Mentha aquatica* and *Typha latifolia,* which prefer to have their roots in highly hydrated soils and sediments (Jäger et al. 2007; Grace 1989). Regarding soil texture preferences, *Carex pendula* and *Mentha aquatica* are more likely to grow in clayey soils, *Typha latifolia* and *Juncus inflexus* in silty soils, while *Salix alba* prefers coarse-textured soils. These habitat conditions and the presence of a significant amount of emerged sediments favoured the settlement of *Salix alba* in this location where it could take roots.

# **Method development**

## **Pesticides extraction from plants: method development**

Organic contaminants have been studied in vegetation for years for food safety or sanitation purposes. Thus, various extraction, purification and quantification methods have been developed. As in our case study, the first challenge for all these studies was to proceed with the extraction of the pollutant, while removing the residues from the matrix, which consists mainly of sugar and chlorophyll. Most studies have used PSA and graphitized carbon black (GCB) for sample clean-up by dispersive solid phase extraction (d-SPE) (Lehotay, Maštovská, and Lightfield 2005; Anastassiades et al. 2003). The use of GCB was validated by Wu et al. (2015)) who found a matrix effect between 10 and 20%, and by Walorczyk et al., (2015) who obtained a recovery rate between 90 and 114% for samples spiked with boscalid, metolachlor, tebuconazole and pendimethalin. In addition, Rutkowska et al., (2018) and Lee et al., (2017) validated this method to analyse other organic contaminants in vegetation with recoveries between 80 and 120%. For the plant matrix clean-up process, we, therefore, consider this method suitable to be developed specifically.

In this study, 1 gram of plant polder was introduced into a 10 mL glass vial and subsequently immersed in 10 mL of methanol (with a purity of 99.8% for residue analysis, sourced from ACROS organics, solubility in methanol is presented in Table SI 1). The mixture underwent vortex mixing for a duration of 5 minutes at a speed of 1200 revolutions per minute (RPM), followed by a 30-minute ultrasound treatment, and another 5-minute vortex mixing session at the same speed. After allowing the samples to decant for 10 minutes, 2.5 mL of the resulting supernatant were carefully extracted and transferred into a 15 mL Falcon® tube.

Subsequently, the purification process employed Primary Secondary Amine (PSA) and graphitized carbon black (sourced from Thermo Scientific) as purification agents through a dispersive solid-phase extraction (d-SPE) method. This purification step aimed to effectively eliminate residual sugar and chlorophyll contaminants from the sample matrix.

Tests were first performed on samples spiked with several combinations of PSA and graphene to obtain the best possible combination while limiting the competition effects with pesticides (see the different combinations in Fig. SI 2, highlighted by the 6 different conditions). The mass of PSA and graphene were chosen according to the methods used by Rejczak and Tuzimski, (2015). The tests were performed as described in Fig. SI 2, all conditions were performed in triplicates.

In the following, only combinations B and E were retained because their chlorophyll content was the lowest. For the evaluation of the matrix effect, 2 mL *2 (replicates) of supernatant were collected and placed in an amber glass bottle. One of these replicates was spiked with 100 µL of a stock solution containing six pesticides (metolachlor, boscalid, epoxiconazole, tebuconazole, aclonifen and pendimethalin) at a final concentration of 100 µg.L^-1^. In the other replicate, 100 µL of methanol was added. In parallel, two replicates containing ultrapure water were prepared as the replicate samples and were used as analytical blanks. For all replicates, 17 mL of ultrapure water was added as well as a 10-mm long stir bars coated with a 0.5 mm film thickness layer of PDMS (purchased from Gerstel), and were stirred for three hours at 1000 RPM for pesticide extraction. Finally, the magnetic bars were collected, rinsed with ultrapure water and dried before pesticide quantification by TD-GC-MS/MS. Two ion transitions (quantification and confirmation) were selected for each pesticide (Table SI 2).

For both conditions, the matrix effects were similar, so condition B (see Fig. SI 2) was chosen as it used less product and was the most economical condition.


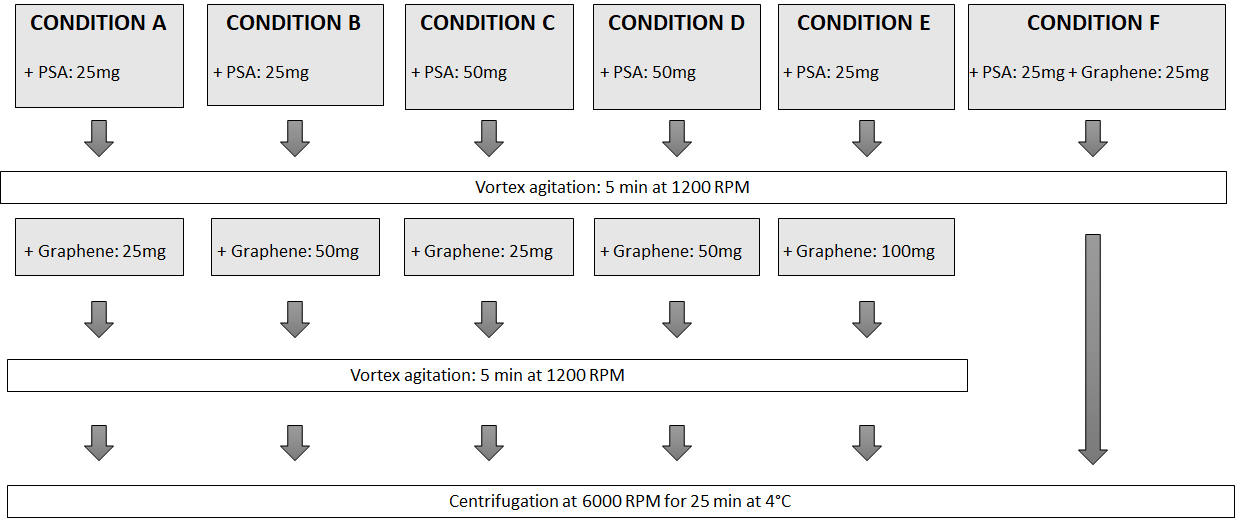
***Fig. SI 2*** *Conditions tested with all combinations of doses*

The analytical performance with extraction condition B was evaluated in terms of trueness and limit of quantification (LOQ). Trueness was evaluated by analysing plant samples spiked at two concentration levels (5 and 20 µg.kg^-1^). Samples were spiked with the target pesticides diluted in 1 ml acetone (purity: 99.9% for residue analysis, ACROS organics) and agitated to ensure that acetone permeated throughout the sample and then placed under a hood for 24 hours to remove it. The next step was to add 10 mL of methanol and 100 µL of an internal standard stock solution (metolachlor D6 at 80 µg.L^-1^, aclonifen D5 at 160 µg.L^-1^, pendimethalin D5 at 80 µg.L^-1^, boscalid D4 at 800 µg.L^-1^ and epoxiconazole D4 at 80 µg.L^-1^, all from Cluzeau Info Labo C.I.L.). These samples were then prepared following the same protocol as described above (with d-SPE performed as condition B), except for the spiking process. For both levels, the recovery for all pesticides was in the range of 71-102% (Table SI 3) and complies with the requirement for analytical methods to be used in the analysis of pesticides. LOQ was estimated as the pesticide concentration with a signal-to-noise ratio of 10 for the transition with the least intensity and from the lowest measured concentration. The quantification limits obtained ranged from 0.01 to 0.39 µg.kg^-1^ (Table SI 3 shows the results).

**Table SI 1 :** Physicochemical parameters of the targeted molecules

| Target analytes | Molecular weight (g.mol^-1^) | CAS No | Log K_OW_ | Solubility in water (mg.L^-1^) | Solubility in methanol (mg.L^-1^) |  |  |  |  |
| --- | --- | --- | --- | --- | --- | --- | --- | --- | --- |
| metolachlor | 283.79 | 1418095-19-8 | 2.9 | 530 | >1000 |  |  |  |  |
| boscalid | 343.21 | 188425-85-6 | 3 | 4.6 | 45000 |  |  |  |  |
| epoxyconazole | 329.76 | 133855-98-8 | 3.3 | 7.1 | >1000 |  |  |  |  |
| tebuconazole | 307.82 | 107534-96-3 | 3.7 | 36 | >1000 |  |  |  |  |
| aclonifen | 264.66 | 74070-46-5 | 4.7 | 1.4 | Miscible* |  |  |  |  |
| pendimethalin | 281.31 | 40487-42-1 | 5.2 | 0.33 | 55000 |  |  |  |  |

*no more specific information was found

**Table SI 2**: Optimised MS/MS parameters (quantifying ion in red, qualifying ion in green, EI quantifying ion in purple)

| Time segments (min) | Pesticide | Retention time (min) | Q1 parent ion m/z | Q3 fragment ion m/z | Collision Energy  (V) | Dwell time  (s) |
| --- | --- | --- | --- | --- | --- | --- |
| 10 | Metolachlor | 13.45 | 238.1 | 162.1 | 10 | 0.1 |
|  |  |  | 162.1 | 133.1 | 13 | 0.1 |
|  | Metolachlor D6 | 13.41 | 242.1 | 166.1 | 10 | 0.1 |
| 13.80 | Pendimethalin | 14.12 | 252.1 | 162 | 9 | 0.1 |
|  |  |  | 252.1 | 191.1 | 7 | 0.1 |
|  | Pendimethalin D5 | 14.08 | 255.1 | 193.1 | 7 | 0.1 |
| 15.50 | Aclonifen | 16.06 | 264 | 194 | 12 | 0.1 |
|  |  |  | 212 | 182 | 10 | 0.1 |
|  | Aclonifen D5 | 16.03 | 269.1 | 217.1 | 10 | 0.1 |
| 16.80 | Tebuconazole | 16.96 | 250.1 | 125.1 | 19 | 0.1 |
|  |  |  | 252.1 | 127.1 | 19 | 0.1 |
|  | Tebuconazole D6 | 16.92 | 256.1 | 125.1 | 19 | 0.1 |
| 17.15 | Epoxiconazole | 17.27 | 192 | 138 | 11 | 0.1 |
|  |  |  | 192 | 111 | 23 | 0.1 |
|  | Epoxiconazole D4 | 17.25 | 196.1 | 142.1 | 11 | 0.1 |
| 18.00 | Boscalid | 20.62 | 342 | 140 | 14 | 0.1 |
|  |  |  | 344 | 142 | 12 | 0.1 |
|  | Boscalid D4 | 20.60 | 346 | 140 | 14 | 0.1 |

***Table SI 3*** *Estimated limits of quantification (LOQ) and trueness expressed as mean recovery*

|  | |  | | | 5 µg/kg | | 20 µg/kg | |
| --- | --- | --- | --- | --- | --- | --- | --- | --- |
| Pesticide | Internal standard | | LOQ (µg/kg) | %RSD^a^ | %R^b^ | %RSD | %R | %RSD |
| metolachlor | metolachlor D6 | | 0.02 | 14 | 99 | 2 | 102 | 4 |
| pendimethalin | pendimethalin D5 | | 0.01 | 27 | 91 | 3 | 97 | 4 |
| aclonifen | aclonifen D5 | | 0.02 | 11 | 96 | 5 | 96 | 6 |
| tebuconazole | epoxiconazole D4 | | 0.07 | 36 | 71 | 1 | 80 | 3 |
| epoxyconazole | epoxiconazole D4 | | 0.02 | 32 | 94 | 2 | 100 | 4 |
| boscalid | boscalid D4 | | 0.39 | 30 | 100 | 13 | 101 | 8 |

^a^ Relative Standard Deviation for three determinations.

^b^ mean recovery (n=3)

## **Interest in SBSE-TD-GC-MS/MS analysis**

Over the last two decades, studies focusing on quantifying organic contaminants in vegetation have mainly used gas chromatography, particularly in food-use vegetables (Lee et al. 2017; Rejczak and Tuzimski 2015; Islam et al. 2018). Our study used stir bar sorptive extraction and thermal desorption coupled with gas chromatography-triple quadrupole mass spectrometry (SBSE-TD-GC-MS/MS (QqQ)) for pesticide analysis. After extraction with methanol, an aliquot was diluted with water, and SBSE is conducted for 180 minutes, using a method introduced by Sandra et al. (2003). To minimize matrix effects, we used PSA and graphitized carbon black for sample clean-up via d-SPE, a novel combination with SBSE. Deuterium-labelled internal standards were used to counteract bias caused by matrix effects. Trueness, calculated in terms of recovery, ranged between 71 and 102% for all pesticides at the two levels studied. Most current studies use gas chromatography with various detection methods, such as ECD, ToF, MSD, and occasionally liquid chromatography, all achieving low limits of quantification (0.3 to 3 µg.kg^-1^). Few methods exist for pesticides in solid samples via SBSE-TD-GC-MS/MS, including the method of Camino-Sánchez et al., (2011) for organic pollutants in marine sediments, producing similar limits of quantification (0.014 µg.kg^-1^ to 1 µg.kg^-1^) to our plant matrix results. This study highlights the usefulness of SBSE-TD-GC-MS/MS for mapping contamination by hydrophilic and hydrophobic pesticides in plant compartments. Additionally, the use of small sample quantities (1 g) and less hazardous solvents makes the method environmentally and ecologically friendly, allowing the quantification of molecules undetectable by other methods, which is of ecotoxicological importance because of the potential deleterious effects of contaminants, even in small quantities.

# **Pesticides stocks**

**Table SI 4** Plant mass repartition in g by compartment and season

|  |  | Plant compartments dry mass (g) | | |
| --- | --- | --- | --- | --- |
|  |  | Roots | Stems | Leaves |
| *Salix alba* | Autumn | 45.5 | 106.9 | 23.4 |
|  | Summer | 25.2 | 40.9 | 13.1 |
| *Carex pendula* | Autumn | 18.4 | - | 67.8 |
|  | Summer | 31.0 | - | 66.0 |
| *Mentha aquatica* | Autumn | 24.8 | 4.8 | 3.3 |
|  | Summer | 13.6 | 6.4 | 6.0 |
| *Typha latifolia* | Autumn | 15.6 | - | 28.0 |
| *Juncus inflexus* | Autumn | 7.5 | - | 46.3 |

Pesticide stocks (in ng) in every compartment (i) was calculated as follows:

${Mass}_{pesticides-compartment i}= {[pesticide]}_{compartment i}* {Mass}_{compartment i}$ (SI 1)

Where ${[pesticide]}_{compartment i}$is the pesticide concentration in the plant compartment i, in µg.kg^-1^ and ${Mass}_{compartment i}$ is in the dry biomass weight of the plant compartment i, in g.

**Table SI 5** Pesticides stocks in the whole considered plants in ng for each season

|  | Autumn 2019 | | | | | Summer 2020 | | |
| --- | --- | --- | --- | --- | --- | --- | --- | --- |
|  | *Salix alba* | *Mentha aquatica* | *Carex pendula* | *Typha latifolia* | *Juncus inflexus* | *Salix alba* | *Mentha aquatica* | *Carex pendula* |
| metolachlor | 33.3 | <D.L | <D.L | <D.L | <D.L | 45.8 | 39.5 | <D.L |
| boscalid | 1371.0 | 710.0 | 512.8 | 436.0 | 377.1 | 448.9 | 432.2 | 1326.5 |
| epoxiconazole | 246.0 | 234.6 | 494.6 | 30.0 | 185.6 | 204.0 | 137.5 | 784.5 |
| tebuconazole | 303.0 | 141.8 | 142.6 | 11.9 | 24.4 | 1070.2 | 129.3 | 602.3 |
| aclonifen | 17.4 | <D.L | <D.L | <D.L | <D.L | 27.9 | <D.L | 27.4 |
| pendimethalin | 178.4 | 2.4 | 8.1 | <D.L | 12.0 | 97.8 | 14.6 | 95.8 |

<D.L: below the detection limit

# **References**

Anastassiades, Michelangelo, Steven J. Lehotay, Darinka Štajnbaher, and Frank J. Schenck. 2003. “Fast and Easy Multiresidue Method Employing Acetonitrile Extraction/Partitioning And.” *Journal of AOAC International* 86: 412–31. https://doi.org/10.2478/s11687-011-0011-9.

Brändel, Markus, and Wolfgang Schütz. 2005. “Temperature Effects on Dormancy Levels and Germination in Temperate Forest Sedges (Carex).” *Plant Ecology* 176 (2): 245–61. https://doi.org/10.1007/s11258-004-0117-y.

Camino-Sánchez, F.J., Alberto Zafra-Gómez, J.P. Pérez-Trujillo, J.E. Conde-González, J.C. Marques, and José Luis Vílchez. 2011. “Validation of a GC–MS/MS Method for Simultaneous Determination of 86 Persistent Organic Pollutants in Marine Sediments by Pressurized Liquid Extraction Followed by Stir Bar Sorptive Extraction.” *Chemosphere* 84 (7): 869–81. https://doi.org/10.1016/j.chemosphere.2011.06.019.

Chaumet, Betty, Jean Luc Probst, Pierre Eon, Thierry Camboulive, David Riboul, Virginie Payré-Suc, Franck Granouillac, and Anne Probst. 2021. “Role of Pond Sediments for Trapping Pesticides in an Agricultural Catchment (Auradé, Sw France): Distribution and Controlling Factors.” *Water (Switzerland)* 1734 (13). https://doi.org/10.3390/w13131734.

Grace, James B. 1989. “Effects of Water Depth on Typha Latifolia and Typha Domingensis Author ( s ): James B . Grace Published by : Botanical Society of America , Inc . Stable URL : Http://Www.Jstor.Org/Stable/2444423 REFERENCES Linked References Are Available on JSTOR for This.” *American Journal of Botany* 76 (5): 762–68.

Islam, Abul Kasem Mohammad Mydul, Su Myeong Hong, Hyo Sub Lee, Byeong Chul Moon, Danbi Kim, and Hyeyoung Kwon. 2018. “Identification and Characterization of Matrix Components in Spinach during QuEChERS Sample Preparation for Pesticide Residue Analysis by LC–ESI–MS/MS, GC–MS and UPLC-DAD.” *Journal of Food Science and Technology* 55 (10): 3930–38. https://doi.org/10.1007/s13197-018-3318-4.

Jäger, A. K., J. P. Almqvist, S. A.K. Vangsøe, G. I. Stafford, A. Adsersen, and J. Van Staden. 2007. “Compounds from Mentha Aquatica with Affinity to the GABA-Benzodiazepine Receptor.” *South African Journal of Botany* 73 (4): 518–21. https://doi.org/10.1016/j.sajb.2007.04.061.

Landgraf, Jessica, Dörthe Tetzlaff, Maren Dubbert, David Dubbert, Aaron Smith, and Chris Soulsby. 2021. “Xylem Water in Riparian Willow Trees (Salix Alba) Reveals Shallow Sources of Root Water Uptake by in Situ Monitoring of Stable Water Isotopes.” *Hydrology and Earth System Sciences Discussions*, no. October: 1–27. https://doi.org/10.5194/hess-2021-456.

Lee, Jonghwa, Leesun Kim, Yongho Shin, Junghak Lee, Jiho Lee, Eunhye Kim, Joon Kwan Moon, and Jeong Han Kim. 2017. “Rapid and Simultaneous Analysis of 360 Pesticides in Brown Rice, Spinach, Orange, and Potato Using Microbore GC-MS/MS.” *Journal of Agricultural and Food Chemistry* 65 (16): 3387–95. https://doi.org/10.1021/acs.jafc.7b00576.

Lehotay, Steven J., Kateřina Maštovská, and Alan R. Lightfield. 2005. “Use of Buffering and Other Means to Improve Results of Problematic Pesticides in a Fast and Easy Method for Residue Analysis of Fruits and Vegetables.” *Journal of AOAC International* 88 (2): 615–29. https://doi.org/10.1093/jaoac/88.2.615.

Norman, Hazel. 2012. “British Ecological Society.” *Encyclopedia of Environmetrics* 29 (2): 369–74. https://doi.org/10.1002/9780470057339.vab033.pub2.

Rejczak, Tomasz, and Tomasz Tuzimski. 2015. “Recent Trends in Sample Preparation and Liquid Chromatography/Mass Spectrometry for Pesticide Residue Analysis in Food and Related Matrixes.” *Journal of AOAC International* 98 (5): 1143–62. https://doi.org/10.5740/jaoacint.SGE1_Rejczak.

Rutkowska, Ewa, Bożena Łozowicka, and Piotr Kaczyński. 2018. “Modification of Multiresidue QuEChERS Protocol to Minimize Matrix Effect and Improve Recoveries for Determination of Pesticide Residues in Dried Herbs Followed by GC-MS/MS.” *Food Analytical Methods* 11 (3): 709–24. https://doi.org/10.1007/s12161-017-1047-3.

Sandra, Pat, Bart Tienpont, and Frank David. 2003. “Multi-Residue Screening of Pesticides in Vegetables, Fruits and Baby Food by Stir Bar Sorptive Extraction–Thermal Desorption–Capillary Gas Chromatography–Mass Spectrometry.” *Journal of Chromatography A* 1000 (1–2): 299–309. https://doi.org/10.1016/S0021-9673(03)00508-9.

Walorczyk, Stanisław, Dariusz Drozdzyński, and Roman Kierzek. 2015. “Two-Step Dispersive-Solid Phase Extraction Strategy for Pesticide Multiresidue Analysis in a Chlorophyll-Containing Matrix by Gas Chromatography-Tandem Mass Spectrometry.” *Journal of Chromatography A* 1412: 22–32. https://doi.org/10.1016/j.chroma.2015.08.022.

Wu, Xiaoli, Runze Zhang, Xiaotong Liu, Wenbi Guan, Xue Liu, Zibo Wang, Yongqiang Ma, and Canping Pan. 2015. “Evaluation of Graphene for Effective Cleanup of Fruit and Vegetable Extracts in Pesticide Residue Analysis.” *Food Analytical Methods* 8 (1): 243–53. https://doi.org/10.1007/s12161-014-9868-9.
